# Ovarian Tissue Cryopreservation versus Other Fertility Techniques for Chemoradiation-Induced Premature Ovarian Insufficiency in Women: A Systematic Review and Future Directions

**DOI:** 10.3390/life14030393

**Published:** 2024-03-15

**Authors:** Eman N. Chaudhri, Ayman Salman, Khalid Awartani, Zaraq Khan, Shahrukh K. Hashmi

**Affiliations:** 1 College of Medicine, Alfaisal University, Riyadh 11533, Saudi Arabia; 2 College of Medicine, Royal College of Surgeons in Ireland, Campus in Bahrain, Busaiteen 15503, Bahrain; 3 Department of Reproductive Medicine, Obstetrics and Gynecolory, King Faisal Specialist Hospital and Research Center (KFSHRC), Riyadh 11211, Saudi Arabia; kawartani@kfshrc.edu.sa; 4 Division of Reproductive Endocrinology and Infertility, Department of Obstetrics and Gynecology, Mayo Clinic, Rochester, MN 55905, USA; 5 Division of Minimally Invasive Gynecologic Surgery, Department of Obstetrics and Gynecology, Mayo Clinic, Rochester, MN 55905, USA; 6 Blood and Marrow Transplant Division, Transplant Center, Mayo Clinic, Rochester, MN 55905, USA; 7 Department of Hematology and Oncology, Sheikh Shakhbout Medical City/Mayo Clinic, Abu Dhabi P.O. Box 11001, United Arab Emirates

**Keywords:** embryo, oocyte, ovarian tissue, chemotherapy, women

## Abstract

Current advances in cancer therapy have increased survival, emphasizing the need for life quality improvement. Fertility loss is common post-chemotherapy. Current guidelines establish embryo and oocyte cryopreservation to address premature ovarian insufficiency (POI). Ovarian tissue cryopreservation has also recently become an acceptable option for fertility preservation, particularly as it is the only option for pre-pubertal patients. Few definitions for optimum fertility outcomes, and few systematic reviews comparing embryo, oocyte, and ovarian tissue cryopreservation as a means of fertility preservation (FP) in pre- and post-pubertal female cancer patients exist. This systematic review aims to improve understanding of gonadotoxic effects of chemoradiation therapy in cancer patients, to analyze the different fertility preservation techniques and procedures available to women with chemoradiation induced ovarian insufficiency, and to compare and recognize the benefits of each technique in restoring fertility, sexual hormone function, and quality of life. Searches were conducted electronically on PubMed, Cochrane, and EBSCOHost, including clinical trials, prospective, and retrospective studies of female cancer patients undergoing anti-cancer therapy, with predefined MeSH terminology. Data were collected, analyzed, and compared. Non-randomized clinical studies were evaluated for risk bias through the Newcastle–Ottawa Scale. In total, 23 studies were included. From there, 647 patients opted for oocyte cryopreservation, 267 for embryo cryopreservation, and 1382 for ovarian tissue cryopreservation (OTC). A total of 175, 18, and 121 live births resulted respectively from oocyte, embryo, and OTC, respectively. Studies without live births discussed other fertility markers as indicators of improvement in sexual hormone function and fertility. The gonadotoxic effects of chemotherapy call for FP intervention. Oocyte and embryo cryopreservation/implantation are well-established procedures. With changing trends and life quality consideration, OTC is a promising interventional method for pre-pubertal patients facing the prospect of fertility loss.

## 1. Introduction

Recent advances in cancer therapy have led to increased cure rates and survival. Consequently, greater emphasis has been placed on improving the quality of life (QoL) of long-term cancer survivors. One such aspect that is being given importance is the possibility of parenthood, which is an important physical, psychological, and social determinant of health. Chemotherapy and radiotherapy are known to result in premature ovarian insufficiency and, thus, loss of fertility [1,2]. Alkylating agents have been implicated in inducing follicular atresia [3]. As the loss of fertility is a major concern for cancer patients, national and international guidelines have been assimilated for management and recommendations for referral to oncofertility interventions before cancer treatment [4,5]. However, most of the data derived from these recommendations come from patients with solid tumors.

According to current guidelines, embryo and oocyte cryopreservation are established interventions for female cancer patients facing the possibility of premature ovarian insufficiency (POI) in the United States (US) [4]. Additionally, ovarian tissue cryopreservation is now considered an acceptable option for fertility preservation and is no longer categorized as experimental in the US [6]. It is the only viable option for pre-pubertal patients and those that cannot delay the start of chemotherapy or radiation [5,7]. 

While these interventions are available, there have historically been few patients referred to specialists for these procedures due to a lack of awareness of available options, and a lack of communication between the multiple specialties that may be involved in a patient’s care [8]. Greater awareness of the progress made in oncofertility may lead to larger numbers of patients being referred for these procedures and avoid the gonadotoxic effects of chemotherapy [9]. Provider bias may also play a part when it comes to patients from lower socioeconomic backgrounds, and perceived future chances of parenthood, as well as inhibitory costs of care/lack of insurance [10]. Establishment of formal programs for fertility preservation have increased the numbers of patients undergoing these procedures, particularly amongst pre-pubertal and female patients [11]. 

There have been many retrospective reviews, prospective studies, and case reports of fertility preservation methods among cancer patients. Varying endpoints have been used to define optimum fertility outcomes in these studies, including ovarian reserve, antral follicle count, numbers of oocytes harvested after ovarian stimulation, hormone levels (particularly anti-Müllerian hormone [AMH] levels), live births, and pregnancy rates. While the physician and scientific communities are particularly interested in the biology and pathogenesis of fertility preservation, from a patient perspective, the most important endpoint or the success of the procedure is live birth rate. The aim of this systematic review is to compare embryonic tissue (ETC), oocyte (OC), and ovarian tissue cryopreservation (OTC) methods by the primary outcome of live birth rates.

## 2. Materials and Methods

All searches were conducted electronically on peer-reviewed databases [12]. Numerous clinical trials and prospective and retrospective studies of patients undergoing fertility preservation procedures including OC, ETC, and OTC were used to conduct this systematic review. 

### 2.1. Study Eligibility Criteria 

All randomized and nonrandomized clinical trials that compared fertility preservation techniques including oocyte, embryo, and/or ovarian tissue cryopreservation were included in data extraction and analysis. Prospective and retrospective cohort studies were included based on the criteria of providing defined successful outcomes and failures with fertility preservation procedures. Case studies and case series with defined success and failure outcomes were also used for data analysis, along with retrospective and prospective studies. Studies with questionnaires were also included in data analysis in fertility preservation efforts which had defined indications of success or failure.

### 2.2. Identification and Selection of Studies 

**Figure 1:**
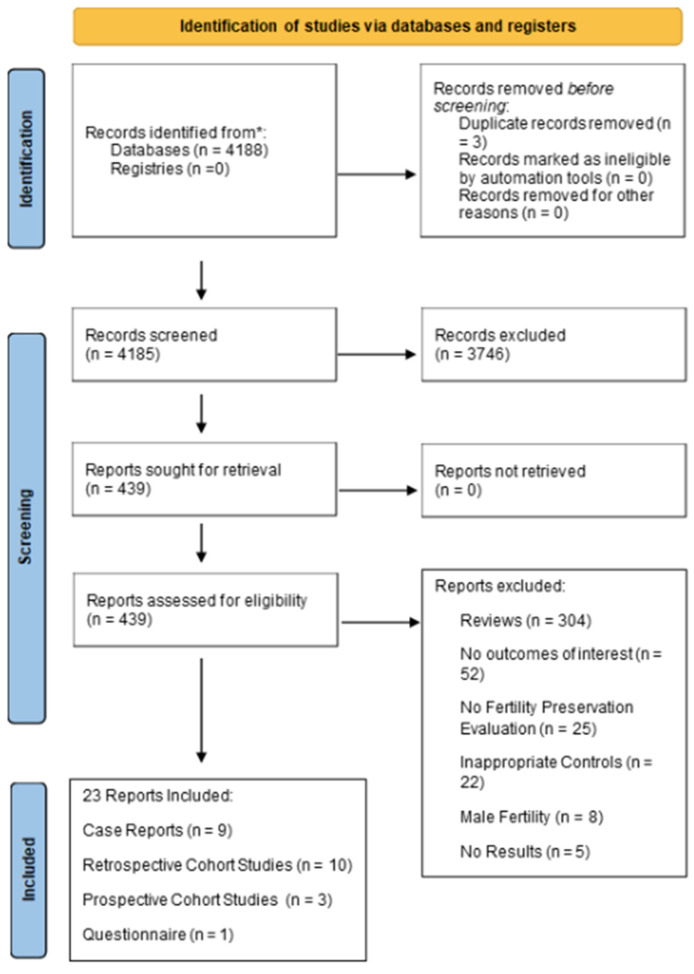
PRISMA 2020 flow chart depicting selection of studies for systematic review. * Databases and Registries include PubMed, Cochrane Library, EBSCOHost, and Google Scholar.

Three databases including PubMed, Cochrane Library, and EbscoHost were searched for fertility preservation with predefined MeSH terminology, including [“oocyte OR embryo OR ovarian tissue cryopreservation AND fertility preservation AND female cancer patients”], in April of 2019. On PubMed, the search initially was not limited to any specific study type. All articles were screened twice manually by two reviewers and included articles were based off reported outcomes of fertility in cancer patients, as shown in the PRISMA 2020 flowchart (Figure 1). Studies were re-screened in June of 2023 to include new publications. Full-text articles and available abstracts (from annual meetings of professional societies) were used for data extraction. The search strategy was peer-reviewed by a fellow author of this manuscript. Reference lists of included studies were also screened. Search results, full-text articles, and available abstracts were independently evaluated by two reviewers. 

### 2.3. Data Collection and Study Appraisal 

We extracted data based on parameters defined by the included studies, regarding cancer patient exposures (chemotherapy, radiotherapy, etc.), doses of chemotherapy or radiation, fertility preservation methods, the average time to conception defined by months after ovarian tissue transplantation, live births, cryopreservation rates, and the number of specified follicles present in ovarian tissue if mentioned. Indications and definitions of success and failure were also assessed in terms of desired outcomes of patients in each study included in the literature review. Relevant sources were used for data extraction including full-text journal articles. 

Study Appraisal: All the included non-randomized clinical trials, including case-control and cohort studies, were evaluated for risk bias through assessment of methodological quality using the Newcastle–Ottawa Scale (NOS) [13]. Case reports were not included in the study appraisal with cohort studies since several of the criteria for validity assessment did not apply to them. Publications were assessed based on selection, comparability, and exposure. In the category of selection, a publication could be awarded a maximum of four points, a maximum of two in comparability, and a maximum of three in exposures, providing an overall maximum possible score of 9. If all categories were awarded few stars, the trial was considered of low quality. As a fair assessment, any trial that was awarded 6 or more points was considered fair or good quality. Data extraction and risk-of-bias assessment were performed for each study in the final sample of studies. 

This study is based on the guidelines of Preferred Reporting Items for Systematic Reviews and Meta-Analyses (PRISMA), and the PRISMA statement is available with the supplementary index. 

## 3. Results

Our search identified a total of 4188 titles and abstracts which were screened. In total, 439 of those full reports were considered relevant to the pertaining systematic review. 

A total of 416 reports were excluded from the articles about the topic of research, mainly due to being formatted as reviews. Other reports did not have outcomes of interest or evaluation of fertility preservation, inappropriate controls, focused on male fertility, had no results, or duplicate records. In total, 23 reports were included in the end, consisting of 9 case reports, 10 retrospective cohort studies, 3 prospective cohort studies, and 1 questionnaire report. No randomized clinical trials (comparing these modalities) were found from any of the database searches. This is depicted in Figure 1, the PRISMA 2020 Flowchart. 

A total of 3271 patients were included in the meta-analysis in the final sample size. All articles focused on female patients, with the exception of 2 which focused on both male and female cancer patients. Studies were conducted in a wide range of countries, the most prevalent being the US. Publication dates of the studies ranged from 2013 to 2021. 

Due to the variability in results and definitions of a successful outcome in the trials in the final selection of studies, we were unable to conduct a meta-regression analysis. In addition to the absence of uniformity in the primary outcome, the study-defined outcomes were mentioned at different time points, thus excluding a random or fixed effects meta-regression. 

OTC, OC, and ETC efforts were evaluated and compared to each other to assess the most effective approach of fertility preservation for female cancer patients facing gonadotoxic anticancer therapy. 

The fertility preservation efforts, as depicted in Tables S1–S3 (see Supplemental Section), respectively, were assessed individually in terms of successful outcomes. Some reports overlapped in the results tables due to having assessed success and failure outcomes for more than one method of fertility preservation.

### 3.1. Ovarian Tissue Cryopreservation (OTC)

**Figure 2:**
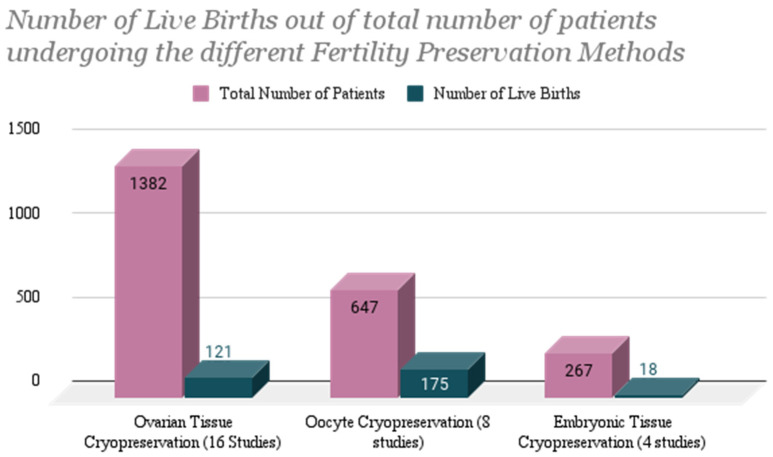
Number of live births out of total number of patients undergoing ovarian tissue cryopreservation re-implantation, oocyte fertilization and implantation, and embryo implantation post-cancer therapy.

A total of 1382 patients from 17 studies explicitly opted for ovarian tissue cryopreservation [3,14,15,16,17,18,19,20,21,22,23,24,25,26,27,28,29]. Out of the 17 studies, a total of 121 live births occurred after re-implantation of ovarian tissue cryopreserved pre-cancer treatment after patients had completed their chemotherapy or radiation therapy (Figure 2). Two of the studies involved patients undergoing both ovarian tissue as well as oocyte cryopreservation [14,16]. Another involved patients opting for more than one of or just one out of the three fertility preservation methods mentioned in the Rodriguez-Wallberg et al. 2019 study [15]. Two of these live births were from overlapping studies: the Lambertini et al. 2018 [16] and the Rodriguez-Wallberg et al. 2019 study [15]. Both live births were specified to be a success from ovarian tissue re-implantation. The third overlapping study, Sigismondi et al. [14], was not applicable to a success rate being measured in pregnancies or live births but focused on ovarian reserve. 

Success rates measured in pregnancies and live births were not applicable for studies with patients who were pre-pubescent at the time of ovarian tissue cryopreservation or re-implantation. The Poirot et al. study [23], involving 418 patients undergoing ovarian tissue cryopreservation, had 0 live births to date, but 84 patients died from their malignancy and were, therefore, not evaluable for fertility preservation outcomes. 

Dolmans et al. analyzed data from 5 major European centers comprising 285 patients who underwent ovarian tissue cryopreservation, with 95 recorded live births [29]. There were 17 recorded live births in the Rodriguez-Wallberg et al. 2016 study [21] out of 46 patients undergoing ovarian tissue cryopreservation, with 1 person undergoing miscarriage. In another study, for 4 live births out of 20 patients who were followed post-chemotherapy and radiation therapy treatments, there was 1 miscarriage [18]. 

Adequate antral follicles and ovarian function were noted in studies of ovarian tissue re-implantation. Oophoropexy was shown to not significantly affect the primordial follicle morphology. Single-site laparoscopies for ovarian tissue procurement were also recorded to be completed successfully with minimal bleeding, a procedure from which 10 successful pregnancies have occurred [26]. 

### 3.2. Oocyte Cryopreservation (OC)

In all studies included in data extraction and analysis, a total of 647 patients in 8 studies explicitly opted for oocyte cryopreservation [14,15,16,30,31,32,33,34]. The total number of live births resulting explicitly from oocyte cryopreservation was 175 (Figure 2). In total, 3 of these studies overlapped with patients who opted for other methods of fertility preservation as well including both embryo cryopreservation and ovarian tissue cryopreservation [15,30,34]. The 3 studies that included both oocyte and embryo cryopreservation resulted in a total of 14 live births (from a total of 359 patients); however, it is unclear which of the two methods the live pregnancies resulted from. In addition, 2 miscarriages occurred, and 5 patients failed to conceive. In one prospective study [15] including 538 patients in which patients opted either for oocyte or embryo cryopreservation, 46% of the patients who opted for oocyte cryopreservation had live births, whereas 54% of the patients who opted for embryo cryopreservation had live births. A total of 164 of these live births came from one study, including 217 patients undergoing chemotherapy for a variety of malignancies [32]. The definition of success in these cases was live births/carrying to term, and the definition of failure was the failure to conceive or carry to term. One study assessing oocyte cryopreservation also conveyed that live births were more likely to occur in patients receiving post-pubertal radiation therapy rather than pre-pubertally [35].

### 3.3. Embryonic Tissue Cryopreservation (ETC) 

Out of all studies included in data extraction and analysis, a total of 267 patients from 4 studies opted explicitly for embryo cryopreservation [15,30,34,36]. The total number of live births from the 4 studies was 18 (Figure 2). There was a total of 2 live births resulting explicitly from transfer of previously cryopreserved embryos, occurring from a case study of a 33-year-old patient who was treated with chemotherapy and radiation therapy for an astrocytoma [36]. Six live births occurred in the Hashimoto et al. study [30] which had patients opting for embryo as well as oocyte cryopreservation and, therefore, the live births were not specified to one specific method of fertility preservation. There was a 20% miscarriage rate recorded in the study. 

All patients in this study were breast cancer patients receiving hormonal therapy (aromatase inhibitors). Eight live births occurred in the Rodriguez-Wallberg et al. study [15], including patients undergoing embryo and ovarian tissue cryopreservation (although the live births were not specified to a specific method of fertility cryopreservation). There were 8 unsuccessful pregnancies, not specific to embryo or ovarian tissue cryopreservation. The Chien et al. study [34] showed a success of 2 live births (twins) from 1 patient out of 20 undergoing embryo cryopreservation. 

## 4. Discussion

When comparing the live birth rate in percentages of the three fertility preservation methods, the highest rate occurred among those who underwent oocyte cryopreservation at a rate of 27% (175 out of 647). Following are patients who underwent OTC at a rate of 8.76% (121 out of 1382), and then those who underwent embryonic tissue cryopreservation at a rate of 6.74% (18 out of 267). The outcomes of OTC, OC, and ETC thus far express that while all work and show promise in preserving fertility and chances at obtaining offspring post-cancer therapy, OC currently yields the highest number of pregnancies and live births, as depicted in Figure 2 and Table 1. The larger number may be attributed to the fact that OC has been in practice for a longer period of time, thus holds more reportable outcomes and promise when presented to patients as a trusted option. 

While ETC outcomes show success, OTC shows the least in comparison to the number of patients with re-implanted tissue. This is likely due to the novelty of the method and the timeframe in which ovarian tissue was extracted from these patients, which was likely at a younger age where OC was not a feasible option and at an age where they would not be ready to try for offspring in the near future. The percentage, therefore, may not accurately depict the success of the method, but rather the result of the context in which the method was conducted, which is on a generally younger population. In comparison, OC has been established for a longer period and, therefore, stands as a more promising method for these patients. ETC also yielded a lower percentage. Like OTC outcomes, this low percentage may not accurately depict the success of the method, but rather the context in which it is performed. Embryonic tissue is fertilized tissue and, therefore, is performed for patients with partners with whom they want to conceive children with. For this reason, the sample size may be smaller to begin with as compared to oocyte and OTC. ETC is also a procedure that has been well established in the past, which may reduce the frequency with which it is mentioned in more recent literature in comparison to newer methods.

While pregnancies and live births were the primary outcomes being assessed in our analysis, it is important to note that other markers of fertility such as ability to conceive, resumption of menstrual cycle, and hormonal levels were also used as outcomes by many studies and, thus, there was a lack of uniformity in result reporting. Table 1 also depicts the unsuccessful outcomes of these methods, but it is important to note that not all methods were carried through for a large number of the sample sizes of the studies due to lack of follow-up and even patient death due to malignancy. 

Gonadotoxic cancer treatments result in substantial numbers of women experiencing POI, which translates into significant distress and lower quality of life in cancer survivors. There are several options that patients may choose to pursue that may allow for fertility preservation. These include ETC and OC, which have become mainstream and established procedures over the years.

OTC, conversely, was regarded as experimental in the USA, until recently when the American Society of Reproductive Medicine deemed it an acceptable form of fertility preservation [6]. Recent evidence suggests that it may be an effective option for some patients, as it is the only suitable option for pre-pubertal patients, and those unable to delay chemotherapy for ovarian stimulation. However, there is a risk of possible reimplantation of malignant cells, thus restricting this option for women with active hematologic malignancies, and possible metastases [37].

Additional procedures that may be considered include gonadotropin-releasing hormone agonists to cause temporary ovarian suppression [32] and ovarian transposition/oophoropexy but data for their uniform application in all cancer patients are conflicting. The suitability of these procedures may vary based on the age of the patient, the type of cancer and subsequent therapy, the ability, and willingness for intra-cytoplasmic sperm injection, and ovarian involvement of cancer [7]. 

The toxic effects of chemotherapy have been long established. Alkylating agents are implicated in infertility, due to the damage induced to granulosa cells surrounding oocytes, as well as their prevention of DNA repair mechanisms [3,38]. Fertility preservation techniques have, therefore, been developed and improved over the years to circumvent the gonadotoxic effects of these cancer treatments. 

Transvaginal aspiration of mature oocytes and subsequent in vitro fertilization for the purposes of embryo cryopreservation is a well-established procedure, with high success rates. However, some drawbacks to this option include the need for controlled ovarian stimulation to stimulate follicular development. Controlled hormonal stimulation of ovaries can take approximately two weeks [39]. However, with the development of newer stimulation protocols, it is no longer necessary to delay hormonal stimulation, as this may be commenced independently of patients’ menstrual cycles [36]. Random start protocols can, therefore, save between 2 and 4 weeks of the overall fertility preservation process [34]. 

Embryo cryopreservation requires fertilization of mature oocytes with the use of a partner’s sperm. In the absence of a partner, donor sperm may be used if acceptable to the patient and if regulations and availability allow for it. To circumvent the immediate need for sperm, oocyte cryopreservation may be performed. Oocyte cryopreservation is also suitable for any post-pubertal patient. With advances in cryopreservation techniques and numerous reports of successful pregnancies, oocyte freezing is not considered an experimental procedure [30]. However, there is still a need for ovarian stimulation and surgical retrieval of oocytes, as is needed for embryo cryopreservation. 

Cryopreservation of ovarian tissue may be achieved by procurement of ovarian tissue (either the whole ovary or cortical strips) via laparoscopy, and then subsequently cryopreserving the tissue as cortical strips for long-term storage [27]. Chemotherapeutic treatment may then commence for the patient. At the desired time of childbearing ovarian tissue may be thawed and re-implanted orthotopically (into the pelvic region) or heterotopically (such as in the abdominal cavity, or the forearm), to allow for ovarian function to resume [39]. 

Internationally, there have been around 130 reported births via ovarian tissue cryopreservation, indicating its potential for success [40]. However, all cancer patients with successful pregnancies because of ovarian tissue cryopreservation were already post-pubertal at the time of cryopreservation. The largest clinical trial involving cancer patients under the age of 15 reported cryopreservation of ovarian tissue for 1031 girls [23]. To date, only three of those patients have undergone re-implantation of their ovarian tissue to induce puberty or restore fertility. No pregnancies have been attempted as per the publication. 

Nevertheless, there have been reports of successful pregnancies for non-cancer patients who had undergone ovarian tissue cryopreservation while pre-pubertal. A 14-year-old girl that had undergone hematopoietic stem cell transplantation (HSCT) due to sickle cell disease requested transplantation of her cryopreserved ovarian tissue. She gave birth to a healthy boy in 2015 [41]. A 9-year-old girl with beta-thalassemia cryopreserved her ovarian tissue prior to HSCT and subsequently gave birth to a healthy baby after transplantation 14 years later [42]. These case reports illustrate the potential success of this intervention particularly amongst pre-pubertal patients facing the prospect of the loss of fertility. 

Future research priorities:

- Comparative retrospective studies in the various methods of fertility preservation, i.e., embryo cryopreservation, oocyte cryopreservation, and ovarian tissue cryopreservation are needed to evaluate outcomes in a uniform population.
- Prospective information regarding markers of ovarian reserve before and after transplantation, as well as utilization of fertility preservation procedures, reasons why there is little uptake of fertility preservation, the utilization of assisted reproductive technology after transplantation, and understanding of the reasons why so few patients who do engage in fertility preservation before transplantation ever opt for using their cryopreserved tissues.
- Novel strategies to prevent or treat chemoradiation-induced ovarian insufficiency including primordial follicle maturation techniques and regenerative medicine (e.g., stromal cells, iPSC) as well as bioengineered ovaries should be tested in clinical trials.
- Though the risk of fertility loss is highest in young patients undergoing HSCT (where typically 12–14 cGys of ionizing total body irradiation is used along with massive chemotherapy), prospective studies in this population are extremely rare. Most of the data comes from case reports and retrospective studies. Thus, prospective trials in HSCT are critically needed.

## Figures and Tables

**Table 1:** Summary of successful and unsuccessful outcomes that have been reported in whole numbers on patients for each fertility preservation method (OTC, OC, ETC) after re-implantation or fertility procedure post-cancer therapy.

	Average Age at Cryopreservation Procedure	Whole Number Reported Successful Outcomes: Live Births (Percentage)	Whole Number Reported Unsuccessful Outcomes: Miscarriages and/or Unsuccessful Conceptions/Implantations
**Ovarian Tissue Cryopreservation**	23.6	8.76%	45 (43 miscarriages, 2 unsuccessful IVF attempts)
**Oocyte Cryopreservation**	31.2	27%	16 (10 miscarriages, 6 unsuccessful conceptions)
**Embryonic Tissue Cryopreservation**	31	6.74%	13 (5 unsuccessful conceptions/implantations, 18 miscarriages)

Key: OTC = ovarian tissue cryopreservation; OC = oocyte cryopreservation; ETC = embryonic tissue cryopreservation.

## Data Availability

Data are contained within the article and Supplementary Materials.

## Supplementary Materials

The following supporting information can be downloaded at: https://www.mdpi.com/article/10.3390/life14030393/s1, Table S1: Ovarian Tissue Cryopreservation, Table S2: Oocyte cryopreservation, Table S3: Embryonic tissue cryopreservation, PRISMA 2020 checklist for systematic reviews.
